# Supplementary material for: Novel vascular plexus in the head of a sea snake (Elapidae, Hydrophiinae) revealed by high-resolution computed tomography and histology
Source: R Soc Open Sci. 2019 Sep 4;6(9):191099. doi: 10.1098/rsos.191099 (PMC6774945; doi:10.1098/rsos.191099)
Supplement: Supplementary material S1 [file rsos191099supp1.pdf]

Royal Society Open Science

Novel vascular plexus in the head of a sea snake (Elapidae, Hydrophiinae) revealed by high-resolution computed tomography and histology.

Alessandro Palci[1,2,*], Roger S. Seymour[3], Cao Van Nguyen[4], Mark N. Hutchinson[1,2], Michael S.Y. Lee[1,2], Kate L. Sanders[3]

[1]College of Science and Engineering, Flinders University, Adelaide, SA 5042, Australia

[2]South Australian Museum, North Terrace, Adelaide, SA 5000, Australia

[3]School of Biological Sciences, University of Adelaide, Adelaide, SA 5005, Australia

[4] Institute of Oceanography, Vietnam Academy of Science and Technology, Hanoi, Vietnam

* Corresponding author: alessandro.palci@flinders.edu.au

Euthanization and fixation protocol:

The specimens of *Hydrophis cyanocinctus* were euthanized with an injection of pure sodium pentobarbital (2 ml) directly into the heart, and their heads were then removed using a sharp blade. The removed heads were then placed in 10% neutral buffered formalin (NBF; 4.0% formaldehyde in phosphate-buffered saline solution) for 2 days, rinsed in a bath of clean water for 1 day, and eventually placed in a small container filled with ethanol (70%) for long-term preservation.

Staining Protocols:

Iodine staining of whole heads:

The protocol used in staining the head of *Hydrophis cyanocinctus* initially followed the recommendations in Gignac et al. (2016) for *Crotalus*, i.e., a staining duration of three weeks, but this resulted in overstaining and eccessive radio-opacity of the sample. The sample was therefore de-stained in ethanol (70%) for about 4 weeks (with one replacement of ethanol after the first 2 weeks) prior to micro-CT scanning for optimal results.
The iodine staining for the specimens of *Hydrophis stokesii* and *Aipysurus laevis* (which were originally preserved frozen, with no fixation) followed an experimentally improved procedure: the specimens were immersed in Lugol's iodine solution (I_2KI, 7.5% concentration) for 1 week, followed by a bath in 70% ethanol for 10 days (with no replacement of the ethanol). We found that this combination of staining and de-staining gives the best results in terms of contrast between different tissues in severed snake heads.

Histology staining:

<u>Alcian Blue/Periodic Acid Schiff</u>: This is a stain for mucins, useful to distinguish between mucous and serous glands. Slides are heated in the oven for 10 minutes

at 65°C; dewaxed with xylene for 6 minutes; washed in absolute ethanol for 2 minutes, in 70% ethanol for 1 minute; and in distilled water for 1 minute; stained with Alcian Blue for 15 minutes; washed in tap water for 2 minutes and then rinsed in distilled water; treated with 1% periodic acid for 5 minutes; washed in distilled water; stained with Schiff's reagent for 10 minutes; washed in running tap water for 5 minutes; nuclei are stained with Lillie–Mayers haematoxylin for 1 minute; washed in running tap water; differentiated with acid alcohol for 3-5 seconds; washed in water; blue nuclei with DAKO Bluing Buffer; washed in water; rinsed in absolute ethanol for 1:00 minutes; placed in xylene for a minute and finally in Histoclear for another minute; coverslipped and dried (rapidly dehydrated, cleared and mounted).

<u>Bielschowsky's modified method for nerve fibers (Biel)</u>: This stain is used to highlight nerve fiber bundles. Sections of the sample (10 µm thick) are heated in the oven for 10 minutes at 65°C; dewaxed with xylene for 6 minutes; washed in absolute ethanol for 2 minutes, in 70% ethanol for 1 minute; and in distilled water for 1 minute; sensitised in 20% silver nitrate for 20 minutes; impregnated in ammoniacal silver solution in a dark room for 15 minutes; 0.5 ml of developer is added to the silver solution and the slides left to rest until the nerve fibers are black and the background is tan; slides are then washed in distilled water, fixed in 5% sodium thiosulphate, rinsed in distilled water; rinsed in absolute ethanol for 2:30 minutes; placed in Xylene for a minute and finally in Histoclear for another minute; coverslipped and dried.

<u>Haematoxylin and Eosin (H&E)</u>: This is a general stain for connective tissues. Slides are heated in the oven for 10 minutes at 65°C; dewaxed with xylene for 6 minutes; washed in absolute ethanol for 2 minutes, in 70% ethanol for 1 minute; and in tap water for 1 minute; stained in DAKO Harris Haematoxylin for 1 minute; rinsed in tap water; stained in DAKO Bluing Buffer for 1 minute; rinsed in tap water for 1 minute and then in 70% ethanol for another minute; stained in DAKO Eosin (Eosin Y Phloxine B) for 4:30 minutes; rinsed in absolute ethanol for 2:30 minutes; placed in xylene for a minute and finally in Histoclear for another minute; coverslipped and dried.

Van Gieson's method for elastic fibers (EVG): This stain is used to highlight blood vessels (elastin) and distinguish between arteries and veins. Slides are heated in the oven for 10 minutes at 65°C; dewaxed with xylene for 6 minutes; washed in absolute ethanol for 2 minutes, in 70% ethanol for 1 minute; and in distilled water for 1 minute; albuminised slides are oxidised with potassium permanganate for 5 minutes; rinsed with distilled water; decolourised with oxalic acid for 5 minutes; washed in distilled water and then in 95% alcohol; stained in Miller's elastic stain for 2 hours; washed in 95% alcohol and rinsed with distilled water; counterstained with filtered Pirofuchsin solution for 3 minutes; blot dried; rapidly dehydrated, cleared and mounted.

Reagents:

Acid Alcohol: add 6.0ml of concentrated Hydrochloric acid to 300ml of distilled water, then add 700ml of absolute alcohol.

Alcian Blue (pH 2.5): dissolve 1.0g of Alcian Blue 8GX in 97 ml of distilled water, add 3 ml of glacial acetic acid, mix well. Filter into the reagent bottle and filter before use.

Ammoniacal silver solution: add drops of strong ammonia (30%) to 20% aqueous silver nitrate until the precipitate dissolves and the solution becomes clear. Add two more drops of strong ammonia in the end.

Developer: 2.5 ml of 20% aqueous citric acid, 2.0 ml of strong (40%) formalin, 1 drop of nitric acid, 95.5 ml of tap water. Made fresh prior to use.

Lillie-Mayers Haematoxylin: purchased from POCD, Cat# - HXIMVS2.5

Miller's elastic staining solution: purchased from Electron Microscopy Sciences, Cat# 26076-10.

Periodic Acid (1.0%): 10ml of 50% periodic acid solution in 500ml of distilled water. Store refrigerated.

Pirofuchsin staining solution: purchased form Merck, Cat# 1.00199.0500.

Schiff's reagent: purchased from Australian Biostain Pty Ltd, PO Box 1407, Traralgon, VIC. Store refrigerated.
