# Supplementary material for: Novel vascular plexus in the head of a sea snake (Elapidae, Hydrophiinae) revealed by high-resolution computed tomography and histology
Source: R Soc Open Sci. 2019 Sep 4;6(9):191099. doi: 10.1098/rsos.191099 (PMC6774945; doi:10.1098/rsos.191099)
Supplement: Supplementary material S2 [file rsos191099supp2.pdf]

Royal Society Open Science

Novel vascular plexus in the head of a sea snake (Elapidae, Hydrophiinae) revealed by high-resolution computed tomography and histology.

Alessandro Palci[1,2,*], Roger S. Seymour[3], Cao Van Nguyen[4], Mark N. Hutchinson[1,2], Michael S.Y. Lee[1,2], Kate L. Sanders[3]

[1]College of Science and Engineering, Flinders University, Adelaide, SA 5042, Australia

[2]South Australian Museum, North Terrace, Adelaide, SA 5000, Australia

[3]School of Biological Sciences, University of Adelaide, Adelaide, SA 5005, Australia

[4] Institute of Oceanography, Vietnam Academy of Science and Technology, Hanoi, Vietnam

* Corresponding author: alessandro.palci@flinders.edu.au

SUPPLEMENTARY FIGURES

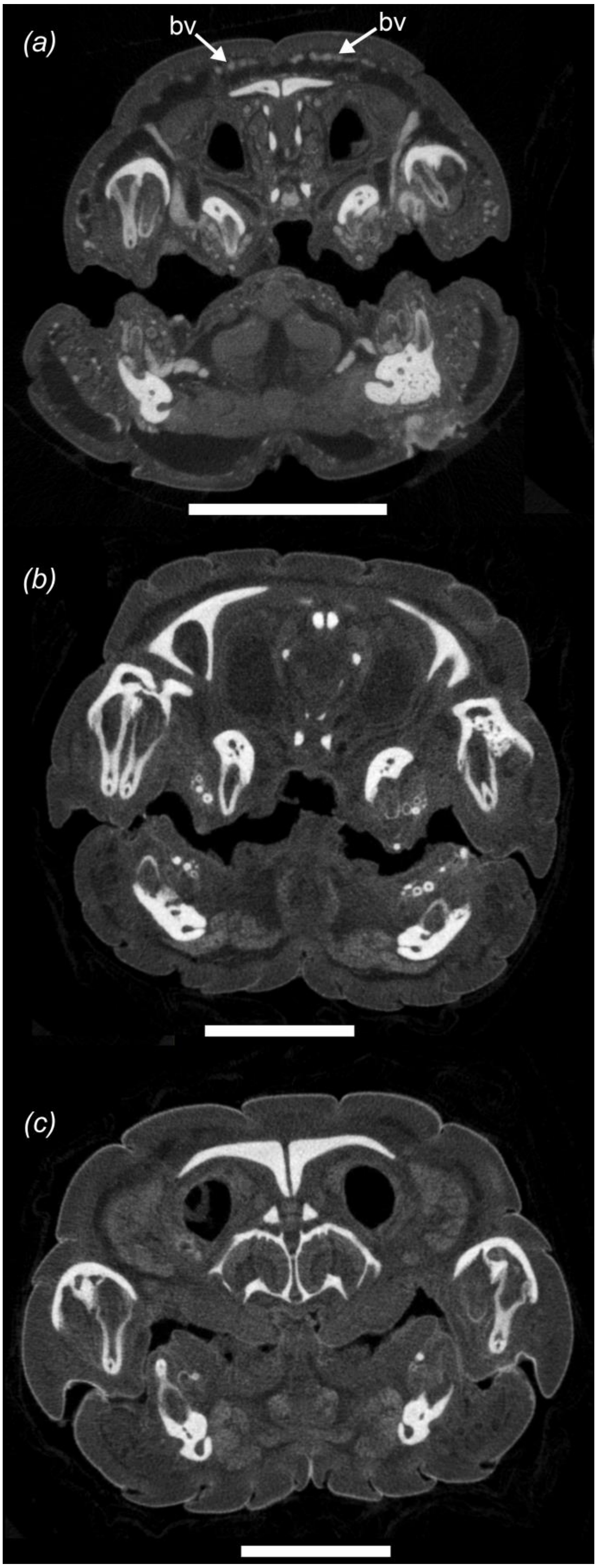

Figure S1. Digitally reconstructed cross sections through the iodine-stained heads of *(a) Hydrophis cyanocinctus*, *(b) Hydrophis stokesii*, and *(c) Aipysurus laevis*. All sections are taken from the snout region, at the level of the maxillary fangs. Note the large blood vessels (bv) under the skin covering the snout in *H. cyanocinctus*, and their absence in the other species. While subcutaneous blood vessels are expected to be present in all three snakes, only those in *H. cyanocinctus* are large enough to be clearly resolved in the CT-scan reconstruction. Scale bar equals 5 mm.

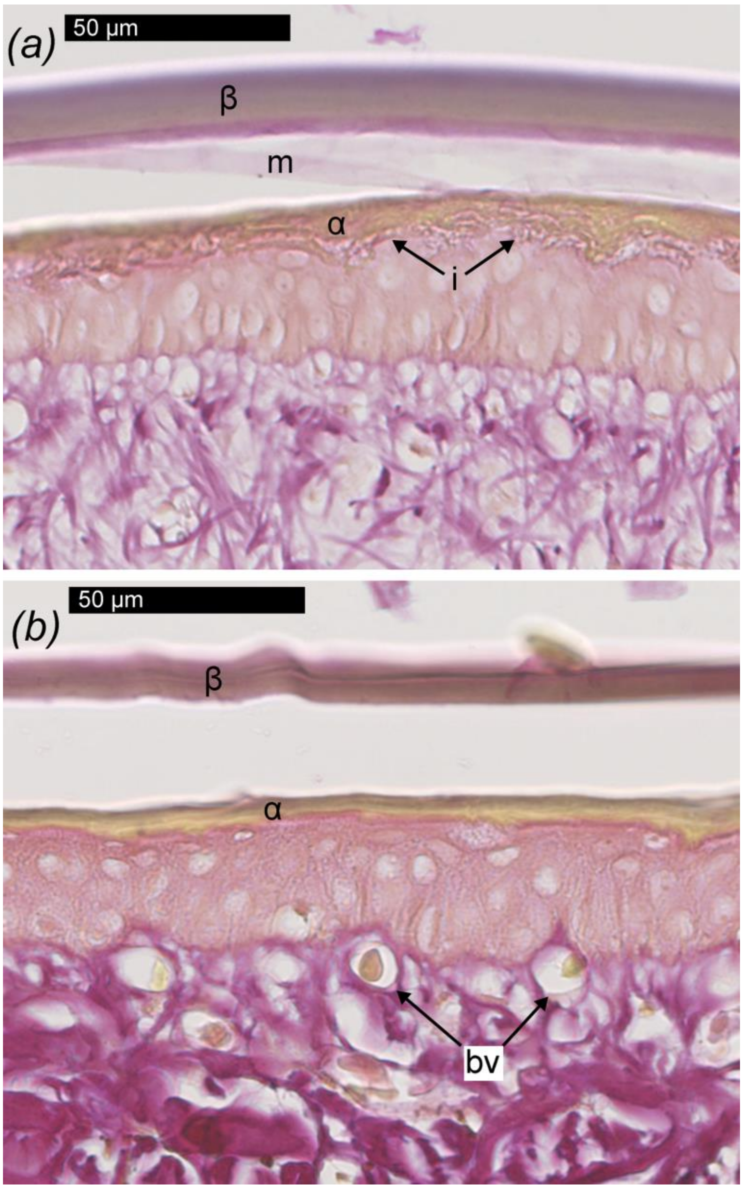

Figure S2. Stained histology sections (Van Gieson’s method for elastic fibers) through the epidermis on the dorsal surface of the head of (a) *Oxyuranus scutellatus* and (b) *Hydrophis cyanocinctus*. Unlike *H. cyanocinctus*, *O. scutellatus* has a distinct mesos layer (m) between the α-layer (α) and the β-layer (β), as well as numerous small inclusions (i) (likely lipid droplets) at the base of the α-layer. Note the small blood vessels (bv) (capillaries) located at the base of the epidermis in *H. cyanocinctus*. The separation of the epidermal layers is an artefact of preparation. Both (a) and (b) are from cross sections through the head.

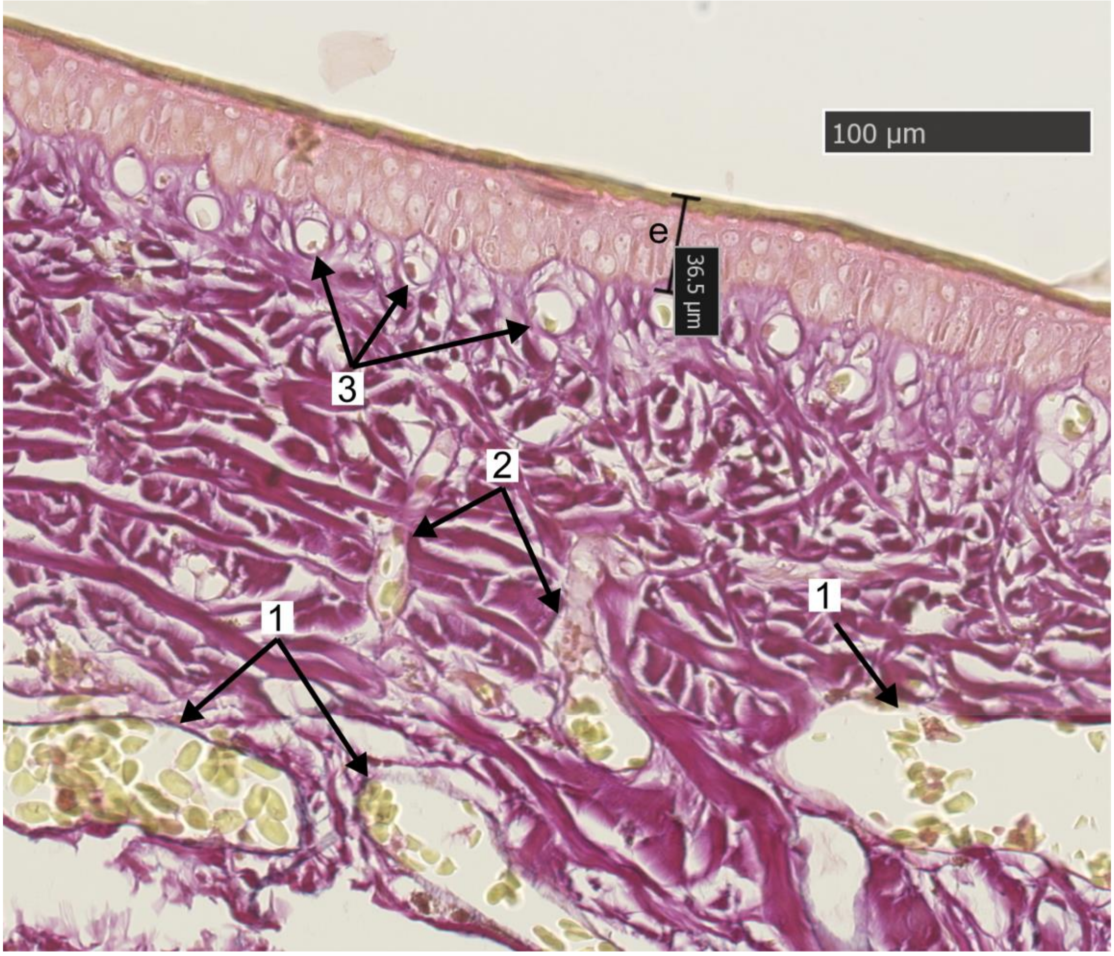

Figure S3. Stained histology section (Van Gieson's method for elastic fibers) through the dermis and epidermis on the dorsal surface of the head of *Hydrophis cyanocinctus*. Note how large veins (1) located at the base of the dermis branch dorsally into thinner blood vessels (2), which then branch into capillaries (3) at the base of the epidermis (e).

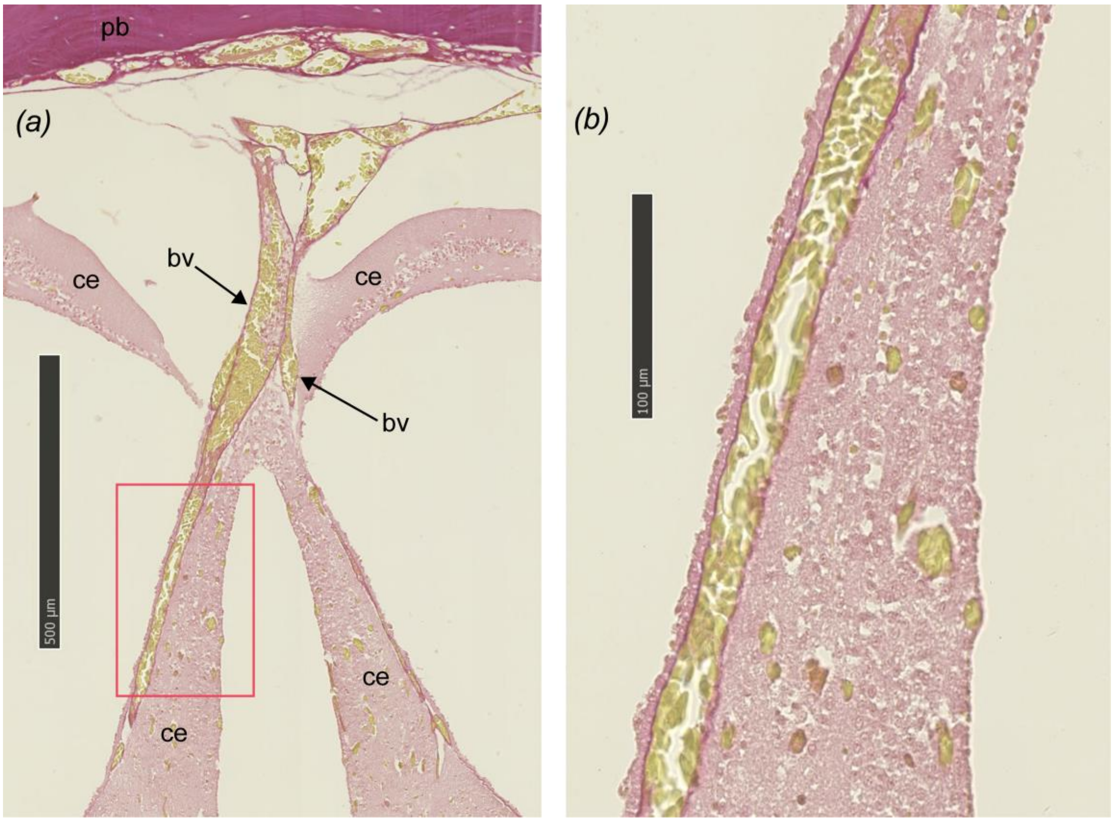

Figure S4. Stained histology section (Van Gieson's method for elastic fibers) through the posterior end of the cerebrum of *Hydrophis cyanocinctus*. Note the pair of large blood vessels in (a) departing ventrally from the dorsal cerebral sinus and entering the brain. (b) Close-up of portion of the left blood vessel highlighted in the red box in (a). The thin tunica media of these blood vessels indicates that they are veins. Erythrocites are stained yellow. Abbreviations: ce, cerebrum; bv, blood vessel; pb, parietal bone.

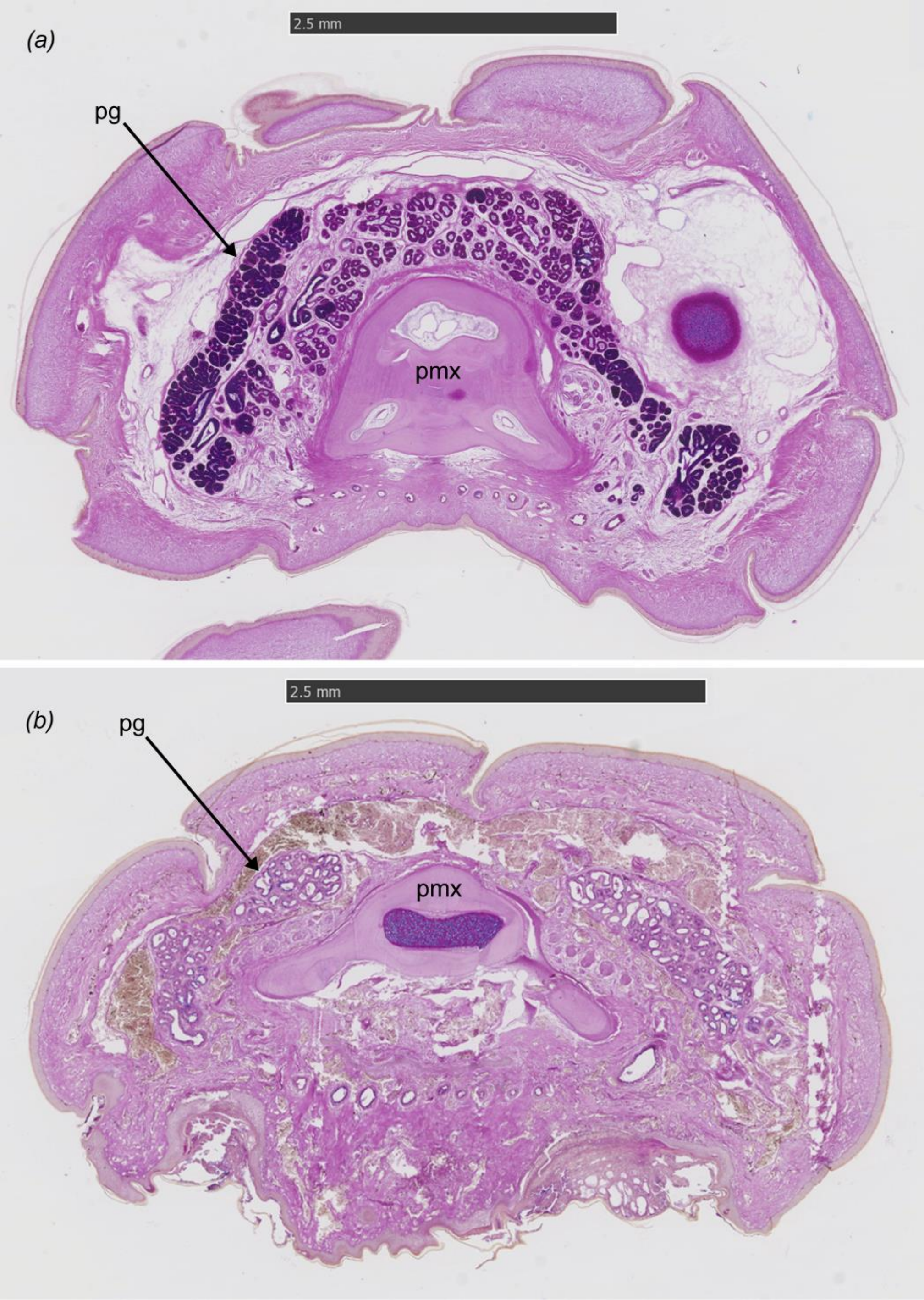

Figure S5. Stained histology sections (Alcian Blue/Periodic Acid Schiff) through the snout of *(a) Oxyuranus scutellatus* and *(b) Hydrophis cyanocinctus*. Note the strong affinity for mucins in the premaxillary gland (pg) of *O. scutellatus* (i.e. the premaxillary gland is mucous), and the much weaker affinity for mucins in the same gland of *H. cyanocinctus* (i.e. the premaxillary gland is mucoserous).

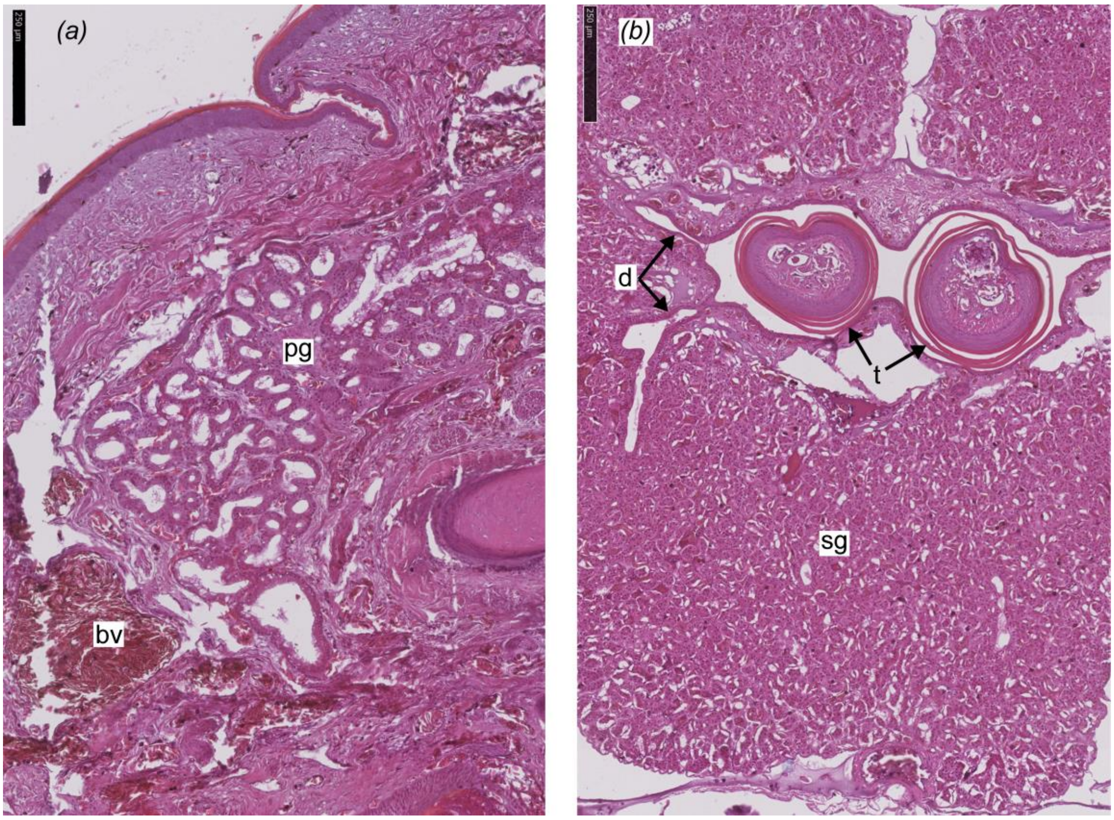

Figure S6. Stained histology sections (haemotoxylin and eosin) through *(a)* the premaxillary gland (pg) of *Hydrophis cyanocinctus*, and *(b)* its sublingual salt gland (sg). Other abbreviations: bv, large blood vessel; d, duct leading from the salt gland into the tongue sheath; t, tongue tines.
